# Cancer screening and follow-up in general practice: A French nationwide cross-sectional study

**DOI:** 10.1080/13814788.2020.1784875

**Published:** 2020-07-17

**Authors:** Marion Lamort-Bouché, Marine Chardon, Nadir Kellou, Isabelle Ray-Coquard, Cyrille Colin, Laurent Letrilliart

**Affiliations:** a Université de Lyon, Lyon, France; b Université de Lyon, Université Claude Bernard Lyon 1, Unité mixte de recherche Epidémiologique et de Surveillance Transport Travail Environnement, Lyon, France; c Département d’Oncologie Médicale, Centre Léon Bérard, Lyon, France; d Unité d'Evaluation Médico-Economique, Pôle Information Médicale Evaluation Recherche, Lyon, France; e Université de Lyon, Université Claude Bernard, Lyon, France

**Keywords:** General practice, cancer screening, cancer survivor, comorbidity, procedure

## Abstract

**Background:**

The overall activity of general practitioners (GPs) related to cancer screening and follow-up is poorly documented.

**Objectives:**

To describe cancer screening and follow-up activities carried out in general practice and analyse them according to the socio-economic characteristics of patients.

**Methods:**

We used data from a French nationwide, multicentre, cross-sectional study that described the distribution of health problems managed in general practice and the associated processes of care. Analyses were adjusted on age and gender when appropriate, using a multivariate, hierarchical, linear mixed-effects model.

**Results:**

Among 20,613 consultations recorded, 580 involved cancer screening (2.8%) and 475 cancer follow-ups (2.3%). The most frequent cancer screening procedures concerned colorectal cancer (38.6% of screening procedures), breast cancer (32.6%), cervical cancer (17.0%), and prostate cancer (9.3%). In consultations with female patients, the most frequent types of cancer followed up were breast (44.9%) and colorectal cancer (10.5%), and with male patients, the most frequent were prostate (37.3%) and skin cancer (10.3%). After adjustment on age and gender, consultations with cancer follow-up included a mean 1.9 health problems managed in addition to cancer. Consultations with cancer screening or follow-up issue less often involved a patient on low income than other consultations (2.4% vs. 4.2%, and 1.1% vs. 4.2%, respectively).

**Conclusion:**

Around 5% of French general practice consultations include cancer screening or follow-up. Socio-economical inequalities demand further research.

KEY MESSAGES

- Five per cent of general practice consultations include cancer screening or follow-up.
- Patients may face economic health inequalities regarding cancer screening and follow-up in general practice.

## Introduction

The incidence of cancer has increased over recent decades [1]. It is expected to keep increasing over time due to the advancing age of the world population, environmental risk factors, and use of cancer screening procedures [2]. Also, advances in cancer treatments are increasing the prevalence of cancer [3], and therefore cancer survivors will face the long-term effects of their disease and its treatments.

In theory, primary care providers have essential roles across the cancer continuum, from screening and early diagnosis to providing care during and after treatment for both cancer and any comorbid condition, and ultimately delivering palliative care at the end of life [4]. French general practitioners (GPs) are directly involved in the screening of several cancers and can be involved in the diagnosis and follow-up of any cancer (Box 1) [5]. In this article, screening refers to ‘the presumptive identification of unrecognised disease or defect by the application of tests, examinations or other procedures which can be applied rapidly’, which covers organised and opportunistic screening [6]. It is intended for a healthy, asymptomatic population. Follow-up refers to care given to a patient over time after finishing specific treatment for cancer. Although various studies have described providing a particular screening test or the follow-up of specific cancer in general practice [7–10], detailed data on the GPs’ overall activity related to cancer screening and follow-up are rare [11,12] despite the need for monitoring the involvement of GPs in cancer control.

BOX 1. Cancer screening and management in the French healthcare system.

**Organised screening [**5**]**
*Breast cancer*: mammography is recommended every two years in women aged 50–74 years. A prescription is sent to the patient’s home. The general practitioner (GP) usually receives the result. The awareness month is Pink October.
*Colorectal cancer*: a faecal immunological test is recommended every two years in patients aged 50–74 years. An invitation to consult the GP is sent to the patient’s home. The GP explains the screening procedure and hands over the equipment. The awareness month is Blue March.
**Opportunistic screening** *Cervical cancer*: a smear is recommended every three years in women aged 25–65 years. Gynaecologists, GPs, and midwives can perform the smear. This screening has become organised in 2019.
*Melanoma*: regular skin examination is recommended in at-risk patients.
*Prostate cancer*: not recommended.
*Lung cancer*: not recommended.
**Cancer treatment and follow-up**
Cancer care is 100% covered by public health insurance through exemption status for the care of chronic severe diseases (‘long-term disease status’). Cancer treatments such as surgery, chemotherapy, radiotherapy, or immunotherapy, are delivered in hospitals. GPs work in private practices. Each patient has a regular physician of his or her choosing. This physician can be involved in cancer diagnosis, follow-up of cancer and its comorbidities during the treatment phase and after, in collaboration with cancer specialists. In particular, the GP can prescribe sick leave and hormone therapy, and provide psycho-social care.

The present study aimed, therefore, to describe the consultations with cancer screening (in asymptomatic patients) or follow-up (after cancer diagnosis) in French general practice and analyse them according to the socio-economic characteristics of patients.

## Methods

### Study design

We used the database constructed for the ECOGEN study, a French, nationwide, multicentre, cross-sectional study conducted in general practice that described the distribution of health problems managed in general practice and the associated processes of care, as a source for secondary analyses [13]. The investigators were 54 interns from 27 medical schools who acted as passive observers on the days of data collection and reported the regular practice of their 128 GP supervisors. They had been trained to identify health problems and associated care processes, and to use the International Classification of Primary Care (ICPC-2) [14].

### Data collection

Over 20 days distributed between December 2011 and April 2012, the interns collected data for each consultation, irrespective of the reason(s) for the encounter. The limited time frame was imposed by the rotation period of the interns in the office of supervising GPs. The following data were collected: consultation locations (office or home visit) and duration; patient age, gender, medical fee exemption status for low income (full financial coverage by the national public healthcare insurance for individuals on low income) as a marker of social inequalities in health; health problem assessments and the care processes (performed or ordered during the consultation) associated with each of these, along with a free-text description. The ECOGEN database recorded only the health problems managed, provided that the patient encounter involved its management through at least one care process. The care processes included various preventive, screening, diagnostic, curative, administrative, and coordinative tasks. Data were collected on a paper case report form (CRF) filled out by the intern at the end of each consultation, and the data collected were later entered into a central database via a dedicated website. The health problem assessments and care processes were classified using the ICPC-2, with the support of an encoding engine system [15]. If a patient refused to participate, they recorded the reason for the refusal.

### Data retrieval and statistical analyses

Data were managed and analysed using SAS software (version 9.4, SAS Institute Inc., Cary, NC, USA). The relational database underwent quality control to detect missing or inconsistent values. Since each procedure code was linked to a health problem assessment code (according to the ICPC-2 classification), we could identify screening procedures as those linked to health problem assessments coded A98 (Health maintenance/prevention). As ICPC-2 codes are sometimes not sufficiently granular, we complemented code retrieval by using the associated free-text description to improve the specificity of the screening procedures and cancer diagnoses. The search algorithms for identifying cancer screening and follow-up situations are presented in Supplementary Appendix 1. Among other health problem assessments managed during the consultation, chronic conditions were determined based on a subclassification of ICPC-2. When the reported consultation duration was longer than one hour, we considered this variable as missing data.

We compared categorical data using Pearson’s Chi-square test, and numerical data using Student’s *t*-test. Also, we compared the number of problem assessments and chronic conditions as well as the duration of the consultation, after adjustment on patient age and gender, using a multivariate, hierarchical, linear mixed-effects model. This model considers the data structure and controls any confounding effect of age and gender. All tests with *p*-values less than 0.05 were considered statistically significant.

### Ethical approval

The ECOGEN study was approved by the national data protection commission (CNIL; No 1549782) and by the regional ethics committee (CPP Sud-Est IV; No.L11-149). Authorisation for the use of ICPC-2 was obtained from WONCA.

## Results

After the exclusion of 168 patients who refused to participate (0.8%), a total of 20,613 consultations were recorded, including 580 with at least one cancer screening (2.8%) and 475 with at least one cancer follow-up (2.3%). When including the 15 consultations involving both cancer screening and follow-up within the same encounter, the overall frequency of cancer screening and/or follow-up was estimated to be 5.0% of consultations (95% CI 4.7–5.3%).

**Figure 1.**
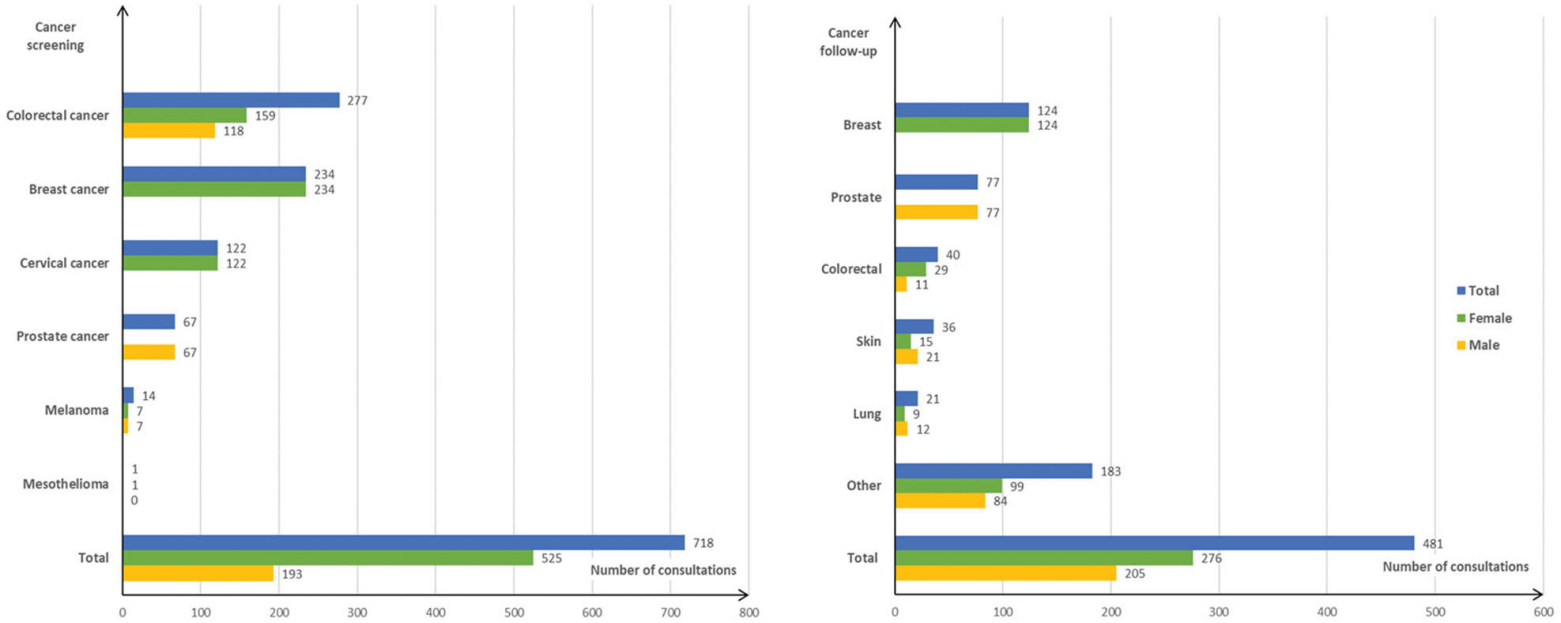
Distribution of cancer screening procedures (*n* = 718 screening procedures in 580 consultations; three missing data) and five most frequent cancers followed up (*n* = 481 cancers followed up in 475 consultations), according to patient gender.

Consultations with cancer screening more often included female patients (67.6% vs. 57.9%) and patients aged between 50 and 74 years (66.9% vs. 32.8%); these patients benefited less frequently from full financial coverage for low income (2.4% vs. 4.2%), and, adjusted on age and gender, the mean duration of the consultation was longer (19.4 vs. 16.6 min) than for those that did not include cancer screening (Table 1). The most frequent cancer screening procedures concerned colorectal cancer (38.6% of screening procedures), breast cancer (32.6%), cervical cancer (17.0%), and prostate cancer (9.3%; Figure 1). Colorectal cancer screening was as frequent in men as in women (1.27% vs. 1.32%, *p* = 0.79). Colorectal, breast, and prostate cancer screening mostly concerned patients aged 50–74 years (84.1%, 56.5%, and 76.1%, respectively), whereas cervical cancer screening mostly concerned patients aged 20–74 years (98.3%). The cancer screening procedure was initiated by the GP, without any request from the patient, in 46.7% of cases (*n* = 335).

**Table 1.** Characteristics of consultations with cancer screening and follow-up, as compared to other consultations.

	Consultations with cancer screening (*n* = 580)	Consultations without cancer screening (*n* = 20,033)	*p*-value	Consultations with cancer follow-up (*n* = 475)	Consultations without cancer follow-up (*n* = 20,138)	*p*-value
Patient gender [*n* (%)]			<0.0001			0.68
Male	188 (32.4%)	8430 (42.1%)		203 (42.7%)	8415 (41.8%)	
Female	392 (67.6%)	11603 (57.9%)		272 (57.3%)	11723 (58.2%)	
Patient age [*n* (%)]			<0.0001			<0.0001
0–19 years	3 (0.5%)	3885 (19.4%)		3 (0.6%)	3885 (19.3%)	
20–49 years	126 (21.7%)	6293 (31.4%)		37 (7.8%)	6382 (31.7%)	
50–74 years	388 (66.9%)	6575 (32.8%)		259 (54.5%)	6704 (33.3%)	
75 years and older	63 (10.9%)	3280 (16.4%)		176 (37.1%)	3167 (15.7%)	
Patient medical fee exemption status for low income^a^ [n (%)]			0.034			0.0001
Yes	14 (2.4%)	843 (4.2%)		5 (1.1%)	852 (4.2%)	
No	566 (97.6%)	19190 (95.8%)		470 (98.9%)	19286 (95.8%)	
Place of consultation [n (%)]			<0.0001			<0.0001
Doctor’s office	571 (98.5%)	18773 (93.7%)		402 (84.6%)	18942 (94.1%)	
Home	9 (1.5%)	1260 (6.3%)		73 (15.4%)	1196 (5.9%)	
Problem assessments^b^, unadjusted Problem assessments^b^, adjusted^c^ [m (95%CI)]	3.68 [3.54–3.81]	2.17 [2.15–2.19]	<0.0001	3.36 [3.20–3.53]	2.18 [2.16–2.20]	<0.0001
	3.43 [3.32–3.54]	2.19 [2.17–2.21]	<0.0001	2.89 [2.77–3.01]	2.19 [2.17–2.20]	0.0045
Chronic conditions^d^ unadjusted Chronic conditions^d^, adjusted^c^ [m (95%CI)]	1.27 [1.16–1.38]	0.84 [0.83–0.86]	<0.0001	1.23 [1.11–1.35]	0.85 [0.83–0.86]	<0.0001
	1.05 [0.97–1.13]	0.88 [0.87–0.90]	0.0001	0.73 [0.64–.082]	0.86 [0.85–0.88]	0.0045
Duration of consultation, unadjusted Duration of consultation, adjusted^c^ [m (95%CI)]	20.08 [19.40–20.78]^e^	16.60 [16.49–16.72]^f^	<0.0001	20.28 [19.42–21.14]^e^	16.62 [16.51–16.73]^f^	<0.0001
	19.39 [18.71–20.07]^e^	16.56 [16.44–16.68]^f^	<0.0001	19.27 [18.52–20.01]^e^	16.58 [16.47–16.69]^f^	<0.0001

m: mean, 95%CI: 95% confidence interval (standard error).

^a^ Full financial coverage by the national public healthcare insurance for individuals with low income.

^b^ Including the cancer assessment.

^c^ Data were adjusted on age category and gender.

^d^ Apart from any cancer.

^e^ Missing data for two consultations.

^f^ Missing data for 112 consultation.

Consultations with cancer follow-up included more often patients older than 50 years of age (91.6% vs. 49.0%), and less frequently patients with full financial coverage for low income (1.1% vs. 4.2%) than other patients (Table 1). In consultations with female patients, the most frequent types of cancer followed up were breast (44.9%) and colorectal cancer (10.5%), and with male patients, the most frequent were prostate (37.3%) and skin cancer (10.3%; Figure 1). For cancer management, GPs most frequently performed medical examination (26.2% of care processes), drug prescription (19.8%), patient education/listening (12.6%), administrative procedures (8.7%), and discussion of test results (6.7%; Table 2). Administrative procedures mainly corresponded to the medical fee exemption status for the cancer disease (34.5%) and sickness certification (26.4%). After adjustment on age and gender, consultations including patients with cancer follow-up had a mean 2.9 health problems managed during the consultation, i.e. 1.9 health problems in addition to cancer (Table 1). The most frequent of these comorbidities were uncomplicated hypertension (25.5% of patients), health maintenance/prevention situation (23.4%), lipid disorder (13.9%), depressive disorder (7.8%), and sleep disturbance (6.7%; Table 3). These health problems managed in consultations with cancer patients were less often chronic conditions than in other consultations (adjusted: 0.73 vs. 0.86), and the mean duration of consultations for such patients lasted longer (adjusted: 19.3 vs. 16.6 min; Table 1).

**Table 2.** Care processes for cancer follow-up (*n* = 1013 for 481 cancers).

	*n* (%)
Medical examination	265 (26.2%)
Medication script/Request/Renew/Injection	201 (19.8%)
Patient education/Listening	128 (12.6%)
Administrative procedure	88 (8.7%)
Results of tests/Procedures	68 (6.7%)
Blood test	61 (6.0%)
Referral to Physician/Specialist/Clinic/Hospital	4 (4.2%)
Results of exam/Test from other provider	37 (3.6%)
Diagnostic radiology/Imaging	35 (3.5%)
Other	87 (8.6%)
Total	1013 (100.0%)

**Table 3.** The 15 most frequent comorbidities managed during consultations with cancer follow-up (*n* = 581 in 475 consultations).

	*n* (% of consultations)
Hypertension, uncomplicated	121 (25.5%)
Health maintenance/Prevention	111 (23.4%)
Lipid disorder	66 (13.9%)
Depressive disorder	37 (7.8%)
Sleep disturbance	32 (6.7%)
Hypothyroidism/Myxedema	30 (6.3%)
Diabetes, non-insulin-dependent	30 (6.3%)
Osteoporosis	29 (6.1%)
Constipation	24 (5.1%)
Hypertension, complicated	23 (4.8%)
Atrial Fibrillation/Flutter	18 (3.8%)
Anxiety disorder/Anxiety state	18 (3.8%)
Upper respiratory infection, acute	16 (3.4%)
Vitamin/Nutritional deficiency	14 (2.9%)
Atherosclerosis/Peripheral vascular disease	12 (2.3%)
Total	581 (122.1%)

## Discussion

### Main findings

Overall, five percent of consultations in French general practice were related to cancer screening or follow-up. Most screening procedures for cancer were performed in consultations with patients aged 50–74 years, and consultations with women included more often screening procedures than did those with men. The most frequent cancers managed by GPs were breast, prostate, colorectal, and skin cancers and they mainly affected patients over 50 years of age. The main processes of care performed for cancer follow-up were medical examination, drug prescription, education/listening, and administrative procedures.

### Strengths and limitations

To the best of our knowledge, this is the first quantitative study of the overall cancer-related activity of GPs based on detailed consultation data. As reported elsewhere, the quality of data entry in the database used herein was assessed through double entry in the database of a subsample of 987 CRFs (4.7%); there was no significant difference in the mean number of problems managed (mean difference: 0.002; *p* = 0.69) but a different code was recorded for 3.2% of the problems [13].

The practices involved were well distributed across metropolitan France, apart from the south-western part of the territory, and this could influence screening and follow-up of skin cancers [13]. It is also of note that the data were collected in the early part of the present decade, but the French screening system has been stable during the last eight years [16]. A possible limitation may also result from the data collection performed in training practices. As published earlier, GPs participating to the ECOGEN study were representative of French GPs for age, gender, practice location, and type of contract with the healthcare system, and the patients of training practices can be considered as broadly representative of patients attending general practice for age [17]. However, a part of the GP supervisors could likely have some specialised clinical activities, e.g. women’s health, but how they compare to other GPs in this regard is not documented. Another point to consider is that the presence of the intern at the consultation could be associated with patient selection; however, their presence was common in these training practices and the intern was a neutral observer, not involved in the care. Finally, the frequency of breast cancer and melanoma screening could be underestimated as the study did not include the awareness month and the summer season, respectively. However, invitations to participate in the organised cancer screenings are sent independently from the breast and colorectal cancer awareness months.

The anonymisation of patient data precluded the identification of those who consulted more than once during the study period. However, the consequences are probably not significant because of the limited time frame for data collection. Indeed, a sensitivity analysis based on removing potential duplicates yielded consistent results (Supplementary Appendix 2).

### Comparison with existing literature

The finding that five percent of consultations involves a cancer-related activity represents approximately one patient per day per GP in France and a substantial proportion of general practice activity that is, by definition, diverse. Cancer screening represents a quarter of the prevention activity of French GPs (that is itself estimated to represent 11% of health issues) and cancer follow-up accounts for approximately 6% of chronic health problems (itself estimated to represent 40% of health problems managed) [13]. The primary screening procedures managed by GPs corresponded to organised screening, and the most frequent was colorectal cancer screening. This is the only organised screening in France that requires consultation in general practice. Also, GPs managed opportunistic screening related to personal risk factors but it is often less evidence-based than organised screening tests, such as for prostate cancer screening. The high frequency of cancer screening procedures initiated by GPs is consistent with the overall high frequency of preventive care initiated by GPs (52.8%) without any request from the patients [13].

Cancer follow-up by GPs was generally consistent with the distribution of the most frequent cancers in the French population, i.e. breast (14.1% of all cancers), prostate (14.0%), lung (11.8%) and colorectal cancers (11.2%) [18]. Whereas lung cancer is the third most frequent cancer, it was the fifth among the cancers followed up in the present study. This may result from the poor prognosis of this cancer (overall survival less than 10% at 5 years) [1], which is likely to be managed primarily in the hospital setting [19]. Conversely, all skin cancers seem relatively frequent in general practice. Although no incidence rate is available in France on skin cancers other than melanoma (3.7%), internationally non-melanoma skin cancers are the fifth most common in terms of incidence [1]. Their high relative survival rate may explain the frequency of their follow-up in general practice.

In the present study, the duration of consultation that included cancer follow-up was longer but fewer numbers of comorbidities were managed. It is, however, known that cancer patients have more comorbidities than other patients [20], and a possible explanation for this is the complexity of cancer management that reduces the time available for the management of other conditions.

We observed that consultations with women included more screening procedures than did those with men, which confirms previous research and surveillance data [21]. This difference may result from a higher rate of primary care consultations by women, from a greater number and earlier screening opportunities (breast, genital tract, colorectal) [22], but probably not from a better willingness to participate in cancer screening since no difference was found for colorectal cancer screening. Despite the French health insurance coverage (medical fee exemption status for low income patients), the results suggest that patients with low income may have fewer screening procedures than others, which may be related to social factors. This is consistent with previous European studies [23], but it is known that deprived people are at higher risk for certain cancers, especially cervical cancer [24]. The study also suggests that cancer patients with low income are less often followed up by their GP than other patients. These patients more often have cancer that has a poor prognosis and multimorbidity [24], which in other studies is found to be a reason to perform follow-up with primary care providers [25].

## Conclusion

Around five percent of French general practice consultations include screening or management of cancer. Cancer, therefore, is a substantial part of the daily work of a GP. Socio-economical inequalities demand further research.

## Supplementary Material

Supplementary Appendix 1

Supplementary Appendix 2

Supplemental Material - STROBE statement
