# Supplementary material for: Cancer screening and follow-up in general practice: A French nationwide cross-sectional study
Source: Eur J Gen Pract. 2020 Jul 17;26(1):95–101. doi: 10.1080/13814788.2020.1784875 (PMC7470121; doi:10.1080/13814788.2020.1784875)
Supplement: Supplementary Appendix 1 [file IGEN_A_1784875_SM0158.docx]

**Supplementary Appendix 1.** Search algorithms for identifying cancer screening and follow up situations.

In ICPC-2, the rubric A98 (Health maintenance/prevention) encompasses health promotion, primary prevention and screening. Cancer screening situations were retrieved from the health problem assessments coded A98 and associated with either: (i) a technical (screening) process (Faeces test [D36], Diagnostic endoscopy [D40], Histological/exfoliative cytology [S37, X37], Blood test [Y34], Diagnostic radiology/ imaging [R41, X41]); (ii) a clinical (screening) examination (for skin cancer [S30, S31], female genital [X30, X31], male genital cancer [Y30, Y31]); or (iii) the combination of an educational process (for digestive system [D45], respiratory system [R45], skin [S45], female genital system [X45], male genital system [Y45]) and a specific free-text description (‘screening, ‘cervical smear’, ‘faecal occult blood test’, ‘mammography’, ‘PSA’, ‘lung X-ray’, ‘clinical breast examination’, ‘digital rectal examination’, ‘skin examination’).

Cancer follow-up situations were retrieved from the health problem assessments corresponding to specific codes (Malignancy not otherwise specified [A79], Hodgkin’s disease/lymphoma [B72], Leukaemia [B73], Malignant neoplasm of blood/other [B74], stomach [D74], colon/rectum [D75], pancreas [D76], digestive system other/not otherwise specified [D77], eye/adnexa [F74], ear [H75], cardiovascular system [K72], musculoskeletal system [L71], nervous system [N74], bronchus/lung [R84], respiratory system/other [R85], skin [S77], thyroid [T71], kidney [U75], bladder [U76], urinary tract [U77], related to pregnancy [W72], of cervix [X75], breast female [X76], female genital/other [X77], prostate [Y77], male genital/other [Y78]), complemented by the associated free-text description.
