# Supplementary material for: Cancer screening and follow-up in general practice: A French nationwide cross-sectional study
Source: Eur J Gen Pract. 2020 Jul 17;26(1):95–101. doi: 10.1080/13814788.2020.1784875 (PMC7470121; doi:10.1080/13814788.2020.1784875)
Supplement: Supplementary Appendix 2 [file IGEN_A_1784875_SM0144.docx]

**Supplementary Appendix 2.** Sensitivity analysis: Characteristics of consultations with cancer screening and follow-up, as compared to other consultations, without potential duplicates (based on age, gender, socio-professional category, student status, and medical fee exemption status).

|  | Consultations with cancer screening  (n=566) | Consultations without cancer screening  (n=15,566) | P-value | Consultations with cancer follow-up  (n=451) | Consultations without cancer follow-up  (n=15,630) | P-value |
| --- | --- | --- | --- | --- | --- | --- |
| Gender [n (%)] |  |  | <0.0001 |  |  | 0.92 |
| Male | 186 (32.9%) | 6723 (43.2%) |  | 195 (43.2%) | 6720 (43.0%) |  |
| Female | 380 (67.1%) | 8843 (56.8%) |  | 256 (56.8%) | 8910 (57.0%) |  |
| Age [n (%)] |  |  | <0.0001 |  |  | <0.0001 |
| 0-19 years | 3 (0.5%) | 2453 (15.8%) |  | 3 (0.7%) | 2453 (15.7%) |  |
| 20-49 years | 123 (21.7%) | 5437 (34.9%) |  | 36 (8.0%) | 5498 (35.2%) |  |
| 50-74 years | 379 (67.0%) | 5303 (34.1%) |  | 249 (55.2%) | 5379 (34.4%) |  |
| 75 years and older | 61 (10.8%) | 2373 (15.2%) |  | 163 (36.1%) | 2300 (14.7%) |  |
| Medical fee exemption status for low incomeᵃ [n (%)] |  |  | 0.0074 |  |  | 0.0002 |
| Yes | 14 (2.5%) | 768 (4.9%) |  | 5 (1.1%) | 773 (5.0%) |  |
| No | 552 (97.5%) | 14798 (95.1%) |  | 446 (98.9%) | 14857 (95.0%) |  |
| Place of consultation [n (%)] |  |  | <0.0001 |  |  | <0.0001 |
| Doctor’s office | 557 (98.4%) | 14621 (93.9%) |  | 386 (85.6%) | 14723 (94.2%) |  |
| Home | 9 (1.6%) | 945 (6.1%) |  | 65 (14.4%) | 907 (5.8%) |  |
| Problem assessments^b^, unadjusted  Problem assessments^b^, adjusted^c^  [m (95%CI)] | 3.67 [3.53-3.81]  3.44 [3.33-3.55] | 2.15 [2.13-2.17]  2.15 [2.13-2.17] | < 0.0001  < 0.0001 | 3.39 [3.22-3.55]  2.94 [2.82-3.07] | 2.17 [2.15-2.19]  2.17 [2.15-2.19] | < 0.0001  < 0.0001 |
| Chronic conditions^d^ unadjusted  Chronic conditions^d^, adjusted^c^  [m (95%CI)] | 1.27 [1.16-1.38]  1.05 [0.97-1.13] | 0.84 [0.82-0.86]  0.85 [0.83-0.87] | < 0.0001  0.0001 | 1.24 [1.12-1.37]  0.77 [0.67-0.86] | 0.84 [0.83-0.86]  0.86 [0.84-0.88] | < 0.0001  0.05 |
| Duration of consultation, unadjusted  Duration of consultation, adjusted^c^  [m (95%CI)] | 20.12 [19.42-20.83]^e^  19.48 [18.80-20.17]^e^ | 16.65 [16.52-16.78]^f^  16.62 [16.49-16.75]^f^ | < 0.0001  < 0.0001 | 20.46 [19.57-21.36]^e^  19.38 [18.61-20.16]^e^ | 16.66 [16.53-16.79]^f^  16.64 [16.50-16.77]^f^ | < 0.0001  < 0.0001 |

m: mean, 95%CI: 95% confidence interval (standard error)

1. Full financial coverage by the national public healthcare insurance for individuals with low income
2. Including the cancer assessment
3. Data were adjusted on age category and gender
4. Apart from any cancer
5. Missing data for 2 consultations
6. Missing data for 112 consultation
